# Yeasts in traditional Baijiu fermentation: diversity, functions, microbial interactions and applications

**DOI:** 10.3389/fmicb.2025.1652173

**Published:** 2025-08-18

**Authors:** Weiwei Dong, Yaxuan Peng, Jiyuan Ma, Yuanliang Hu, Shenxi Chen, Shumiao Zhao

**Affiliations:** ^1^Hubei Key Laboratory of Edible Wild Plants Conservation and Utilization, College of Life Sciences, Hubei Normal University, Huangshi, China; ^2^Hubei Key Laboratory of Quality and Safety of Traditional Chinese Medicine Health Food, Jing Brand Co., Ltd., Daye, China; ^3^National Key Laboratory of Agricultural Microbiology and College of Life Science and Technology, Huazhong Agricultural University, Wuhan, China

**Keywords:** Baijiu, yeasts, non-*Saccharomyces* yeasts, community structure, flavor metabolism, interactions, functions

## Abstract

Baijiu is a traditional distilled liquor unique to China. Its distinctive flavor is shaped by the synergistic activity of complex microbial communities, among which yeasts play a central role in sugar metabolism, ethanol fermentation, and aroma synthesis. In recent years, the advancement of isolation and cultivation techniques, high-throughput sequencing, metagenomics, and multi-omics technologies has deepened our understanding of yeast community compositions, succession patterns, and functional characteristics during Baijiu brewing. Among these, *Saccharomyces cerevisiae* was recognized as the core ethanol-producing species and has been extensively studied for its metabolic traits and stress tolerance in Baijiu fermentation. Studies have shown that, in addition to *S. cerevisiae*, non-*Saccharomyces* yeasts such as *Pichia*, *Wickerhamomyces*, *Saccharomycopsis*, *Kazachstania*, and *Candida* et al. are widely distributed across strong-, sauce-, and light-flavor Baijiu and their respective starters (Daqu), exhibiting robust ester-producing capacities and stress resistance. These yeasts occupy distinct ecological niches throughout fermentation stages and engage in dynamic and environment-dependent interactions with lactic acid bacteria, molds, and other microbes. This review systematically summarizes yeast diversity, community structure, metabolic traits, key functional genes, microecological interactions, recent discoveries of novel yeast species, and advances in genetic engineering in Baijiu brewing. It further highlights future research priorities, including multi-omics integration, functional exploration of non-*Saccharomyces* yeasts, and synthetic biology-guided strain development, with the goal of supporting high-quality and intelligent Baijiu production.

## Introduction

Baijiu is one of the six major distilled liquors globally. Its distinctive aroma types, diverse flavor profiles, and deep cultural heritage confer it a significant role in the global alcoholic beverage market. Baijiu production is characterized by traditional Chinese techniques, using solid-state fermentation with Daqu or Xiaoqu as saccharifying and fermenting starters. The production process includes starter making, fermentation, distillation, and aging. Among these, traditional solid-state fermentation is central, featuring dynamic microbial succession and metabolism on solid substrates (grains), forming a unique microecosystem that underlies Baijiu’s characteristic flavor profile.

**Figure 1:**
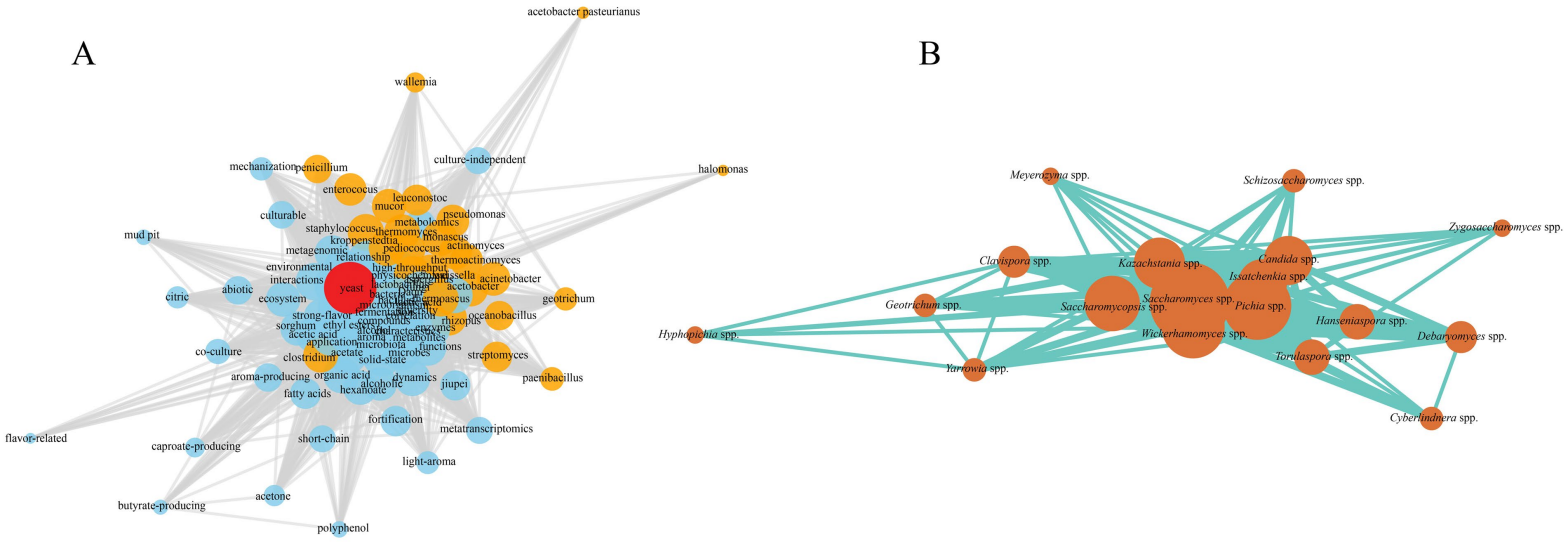
Keyword co-occurrence network highlighting yeast-related research themes in Baijiu studies **(A)** and species-level co-occurrence network of yeasts identified in Baijiu fermentation literatures **(B)**.

Over the past two decades, rapid progress in sequencing technologies has yielded deeper insights into microbial community structure, succession dynamics, metabolic functions, and microbial interactions during traditional Baijiu fermentation. To identify research hotspots, we searched the Web of Science database using “Baijiu” as a keyword and retrieved 2,616 publications from the past 20 years. Keyword analysis and co-occurrence network construction using VOSviewer revealed “yeast,” “microorganism,” “microbial community,” “metabolism,” and “flavor compounds” as central research themes. Among these, yeast emerged as one of the most frequently investigated microbial groups (Figure 1A), underscoring its pivotal role in traditional solid-state Baijiu fermentation.

In this system, yeasts serve not only as the primary ethanol producers during alcoholic fermentation but also play critical roles in synthesizing various flavor compounds. Early researches primarily focused on the metabolic traits of *Saccharomyces cerevisiae* and established it as the core yeast species in Baijiu production. However, with the integration of amplicon sequencing, metagenomics, cultivation techniques, and multi-omics techniques, it has become evident that non-*Saccharomyces* yeasts are also ubiquitous in traditional fermentation ecosystems. These include *Candida* spp., *Pichia* spp., *Kazachstania* spp., *Wickerhamomyces* spp., and *Issatchenkia* spp. et al. (Figure 1B). These non-*Saccharomyces* yeasts occupy specific ecological niches at different fermentation stages and interact with molds, *Bacillus*, lactic acid bacteria (LAB), actinomycetes, and other microbes to co-participate in key metabolic processes including starch degradation, glycolysis, ethanol production, organic acid metabolism, and flavor substance generation.

Furthermore, yeast community structure and function vary substantially across three basic Baijiu types (strong-, sauce-, light-flavor) and their corresponding Daqu (medium-, high-, and low-temperature types). In strong-flavor Baijiu fermentation, *S. cerevisiae* is dominant, but non-*Saccharomyces* yeasts such as *Pichia kudriavzevii* and *Wickerhamomyces anomalus* also contribute to ethanol and flavor production at particular stages (Hu X. et al., 2020; Guan et al., 2020). Especially during late stage of fermentation, acid- and ethanol-tolerant non-*Saccharomyces* yeasts become predominant, displaying clear succession dynamics (Dong et al., 2024a). In sauce-flavor Baijiu, characterized by high temperatures and anaerobic conditions, thermotolerant yeasts such as *Pichia* spp., *Saccharomyces* spp., and *Candida* spp., gradually dominate, cooperating with other microbes in substrate breakdown and flavor production (Li et al., 2022; Hao et al., 2021). In light-flavor Baijiu, typically fermented in ground-based pits, yeast communities show distinct spatial and temporal patterns. Non-*Saccharomyces* yeasts like *Pichia fermentans*, *W. anomalus*, and *Hanseniaspora uvarum* dominate specific stages, contributing to characteristic ester formation such as ethyl acetate and ethyl lactate (Cai et al., 2022; Huang et al., 2020; Luo et al., 2023).

From a microecological standpoint, yeasts form mutualistic relationships with LAB and molds. Yeast-derived metabolites like ethanol and organic acids support their own growth and provide substrates or adjust pH to facilitate LAB proliferation (Chen et al., 2022; Kang et al., 2024). Conversely, LAB suppress spoilage microbes by acidifying the environment, thereby stabilizing yeast populations. Such interactions may transition between mutualism and competition depending on physicochemical conditions such as pH, temperature, and substrate gradients, reflecting the adaptive flexibility of the fermentation microbiome. This “co-metabolism–competition–mutualism” model maintains ecological balance and enhances flavor compound accumulation. For instance, *S. cerevisiae* dominates ethanol production in early to mid-fermentation, while non-*Saccharomyces* yeasts such as *Pichia* spp. and *Candida* spp. produce medium-chain fatty acids, alcohols, and esters in late stages, enriching the complex Baijiu aroma. With advances in omics, novel yeast species have been identified from Baijiu and starter environments. For example, *Starmerella fangiana* f.a. sp. nov., isolated from Daqu-making regions, exhibits strong capacity for ester and medium-chain fatty acid synthesis (Wei et al., 2024). Genetic engineering has further enhanced yeast functionality, including overexpression of *EHT1* to boost ethyl caproate production and construction of high ethyl acetate-producing *S. cerevisiae* strains via CRISPR/Cas9 (Chen et al., 2014; Sun et al., 2025).

Despite these advances, challenges persist: amplicon sequencing lacks species-level resolution, functional studies on non-*Saccharomyces* yeasts are limited, and omics datasets remain fragmented. This review thus comprehensively summarizes yeast diversity, community composition, metabolic function, ecological interactions, and engineering potential in strong-, sauce-, and light-flavor Baijiu and their associated Daqu. Special emphasis is placed on yeast functional roles and cooperative networks, offering theoretical and practical guidance for targeted yeast application in Baijiu fermentation.

## Diversity and community composition of yeasts

In traditional Baijiu brewing systems, yeasts are one of the core microbial groups. They not only dominate the alcoholic fermentation process but also work synergistically with other microbes to synthesize flavor compounds and maintain ecological stability (Ma et al., 2024). In recent years, the integration of technologies such as denaturing gradient gel electrophoresis (DGGE), high-throughput sequencing (HTS), quantitative PCR (qPCR), metagenomics, transcriptomics, and culturomics has gradually elucidated the taxonomic composition, spatial distribution, dynamic succession, metabolic functions, and ecological interactions of yeast communities in Baijiu fermentation systems (Fan et al., 2023; Qin et al., 2024). Especially in studies of different Baijiu aroma types (strong-, sauce-, and light-flavor Baijiu) and their corresponding Daqu, yeast community structure and function have been shown to vary significantly depending on fermentation temperature, brewing processes, production cycle, environmental factors, and geographical conditions (Yao et al., 2022; Li et al., 2024). This section provides a detailed review of the composition, succession patterns, functional differences, and interactions of yeasts in strong-flavor, sauce-flavor, and light-flavor Baijiu and their associated Daqu.

### Yeasts in strong-flavor Baijiu

Strong-flavor Baijiu is the most widely produced and consumed Baijiu type in China, with well-known brands including Wuliangye, Luzhou Laojiao, and Yanghe. Its brewing processes are characterized by traditional crafts of “mud-pit solid-state fermentation” and “usage of medium-temperature Daqu.” The unique fermentation environment of the pit, characterized by high moisture, strong reductivity, elevated acidity, and prolonged fermentation cycles, creates optimal ecological conditions for fostering yeast diversity and facilitating its functional manifestation (Liu et al., 2021; Liu X. et al., 2023). The yeasts in strong-flavor Baijiu primarily originate from Daqu, the fermentation environment, pit mud microecology, raw materials, and brewing equipment, forming a community structure characterized by “multi-source input then dynamic succession” (Hu X. et al., 2020; Guan et al., 2020; Zhang et al., 2021).

Traditionally, *S. cerevisiae* has been considered the predominant yeast species in strong-flavor Baijiu fermentation due to its strong glycolytic capacity and ethanol-producing efficiency, and it plays a critical role in ethanol accumulation and aroma formation (Dong et al., 2024a; Duan et al., 2024; Qiu et al., 2024a). However, recent studies have revealed the widespread presence of non-*Saccharomyces* yeasts in the system, including *P. kudriavzevii*, *W. anomalus*, *Kazachstania exigua*, *Clavispora lusitaniae*, *Issatchenkia orientalis*, *Zygosaccharomyces bailii*, *Candida ethanolica*, and *Torulaspora delbrueckii* (Table 1) (Duan et al., 2024; You et al., 2021; Liu et al., 2021). Despite differences in abundance, these non-*Saccharomyces* yeasts exhibit diverse metabolic capacities and ecological roles, which vary significantly at the species level. For instance, *P. kudriavzevii* is known for its high production of acetic acid and ethyl acetate (Dong et al., 2024a), contributing to fruity and solvent-like aroma notes depending on concentration. In contrast, *K. exigua* is primarily involved in acid regulation through lactic acid production, thereby modulating environmental pH and shaping microbial succession (Dong et al., 2024a). Such genus- and species-specific functional niches extend beyond broad labels like “ester producers” and highlight the intricate division of labor among non-*Saccharomyces* yeasts in strong-flavor Baijiu fermentation. These functional specializations ultimately enhance microbial synergy and contribute to the complexity and stability of the fermentation system (Hu X. et al., 2020; You et al., 2021; Liu et al., 2021; Pang et al., 2023).

**Table 1:** Comprehensive summary of yeast taxonomic composition and functional traits in the brewing of strong-, sauce-, and light-flavor Baijiu and their associated Daqu.

Category	Yeast genus	Dominant yeast genus and abundance	Yeast species	Dominant yeast species and abundance	Functional traits at species level	References
Strong-flavor Baijiu	*Saccharomyces, Pichia, Wickerhamomyces, Kazachstania, Clavispora, Issatchenkia, Zygosaccharomyces, Hanseniaspora, Candida, Torulaspora*	*Saccharomyces (~30%), Pichia (~16%), Wickerhamomyces (10–20%), Kazachstania (~20%)*	*S. cerevisiae, P. kudriavzevii, W. anomalus, K. exigua, C. lusitaniae, I. orientalis, Z. bailii, C. ethanolica, and T. delbrueckii*	*S. cerevisiae (~30%), P. kudriavzevii (~20%), W. anomalus (10–15%), K. exigua (12–18%)*	*S. cerevisiae: High ethanol and ethyl acetate production; P. kudriavzevii: Enhances flavor complexity, acid tolerance; W. anomalus: Antimicrobial and ester synthesis; K. exigua: Acid prodution and yeast regulation*	Duan et al. (2024), You et al. (2021), Liu et al. (2021), Hu X. et al. (2020), Pang et al. (2023), Qiu et al. (2024a), Dong et al. (2024a)
Strong-flavor Daqu	*Saccharomyces, Pichia, Wickerhamomyces, Issatchenkia, Geotrichum, Saccharomycopsis, Yarrowia, Hanseniaspora, Kazachstania*	*Saccharomyces (~25%), Pichia (~20%), Wickerhamomyces (~40%)*	*S. cerevisiae, P. kudriavzevii, W. anomalus, I. orientalis, P. fermentans, G. candidum, S. fibuligera, Y. lipolytica, H. uvarum, and K. barnettii*	*S. cerevisiae (~25%), P. kudriavzevii (~10–15%), W. anomalus (~40%)*	*S. cerevisiae: Ethanol producer, glucose conversion; P. kudriavzevii: Contributes to organic acid metabolism and esters; W. anomalus: Produces ethyl acetate and enhances aroma complexity*	Pu et al. (2021), Yan et al. (2019a), Gou et al. (2015), Ren et al. (2024)
Sauce-flavor Baijiu	*Saccharomyces, Pichia Issatchenkia, Wickerhamomyces, Schizosaccharomyces, Zygosaccharomyces, Candida, Kazachstania*	*Saccharomyces (~10%), Pichia (~35%), Zygosaccharomyces (~25%), Schizosaccharomyces (~15%)*	*S. cerevisiae, P. kudriavzevii, I. orientalis, W. anomalus, S. fibuligera, Z. bailii, C. humilis, and K. exigua*	*S. cerevisiae (~10%), P. kudriavzevii (~28), Z. bailii (~25%), S. pombe (~15%)*	*S. cerevisiae: Ethanol producer; involved in ester and higher alcohol formation; P. kudriavzevii: Ethanol, and ethyl acetate production; Z.bailii: Highly acid- and ethanol-tolerant; involved in lactic acid and acetic acid regulation; S. pombe: contributes to acidity balance*	Chen et al. (2025), Meng et al. (2015), Wu et al. (2012), Song et al. (2017), Li et al. (2022), Sun et al. (2012), Song et al. (2020)
Sauce-flavor Daqu	*Saccharomyces, Pichia, Wickerhamomyces, Saccharomycopsis, Hanseniaspora, Candida, Debaryomyces, Zygosaccharomyces*	*Saccharomyces (~6%), Pichia (~20%), Wickerhamomyces (~25%), Candida (~15%)*	*S. cerevisiae, P. kudriavzevii, W. anomalus, S. fibuligera, H. uvarum, C. stellata, D. hansenii, and Z. bailii*	*S. cerevisiae (~6%), P. kudriavzevii (~16%), W. anomalus (~25%), C. stellata (~15%)*	*S. cerevisiae: Produces ethanol and esters; P. kudriavzevii: Ethyl acetate production; W. anomalus: Ethyl acetate producer, aroma enhancer; C. stellata: Contributes to flavor complexity*	Cai et al. (2021), Fan et al. (2024), Liu et al. (2012), Wu et al. (2023), Yi et al. (2019)
Light-flavor Baijiu	*Saccharomyces, Pichia, Wickerhamomyces, Candida, Issatchenkia, Kazachstania, Torulaspora*	*Saccharomyces (~30%), Wickerhamomyces (~30%), Candida (~10%)*	*S. cerevisiae, P. kudriavzevii, W. anomalus, C. tropicalis, I. orientalis, K. exigua, and T. delbrueckii*	*S. cerevisiae (~30%), W. anomalus (~30%), C. tropicalis (~10%)*	*S. cerevisiae: Produces ethanol and esters, key fermentative yeast; W. anomalus: Ethyl acetate producer, aroma enhancer; C. tropicalis: Contributes to flavor complexity*	Cai et al. (2022), Chen et al. (2022), Wang and Xu (2019), Li et al. (2011), Huang et al. (2020), Xue et al. (2023)
Light-flavor Daqu	*Saccharomyces, Pichia, Wickerhamomyces, Candida, Saccharomycopsis, Issatchenkia, Meyerozyma*	*Saccharomyces (~35%), Wickerhamomyces (~15%), Candida (~20%)*	*S. cerevisiae, P. kudriavzevii, W. anomalus, C. guilliermondii, S. fibuligera, I. orientalis, and M. guilliermondii*	*S. cerevisiae (~35%), W. anomalus (~15%), C. guilliermondii (~20%)*	*S. cerevisiae: Participates in ethanol and ester formation; W. anomalus: Enhances aroma via ester and higher alcohol production; C. guilliermondii: Involved in glucose fermentation and ethyl acetate production*	Zheng et al. (2012), Hu et al. (2021), Li R. Y. et al. (2018), Shi et al. (2009), Xiang et al. (2023)

The yeast community in strong-flavor Baijiu displays distinct stage-dependent succession patterns. Duan et al. (2024) observed that non-*Saccharomyces* yeasts such as *Kazachstania*, *Issatchenkia*, *Pichia*, *Clavispora*, and *Geotrichum* dominated throughout the fermentation process, while *S. cerevisiae* appeared transiently around day 10, temporarily establishing dominance before being re-displaced by non-*Saccharomyces* species, forming a pattern that non-*Saccharomyces* became dominant, then *S. cerevisiae* was transient advantage, and non-*Saccharomyces* reoccupied. This succession pattern closely correlated with changes in pH, temperature, and reducing sugar levels. You et al. (2021) reported that *K. exigua* dominated in early fermentation, modulating environmental acidity via lactic acid production and influencing subsequent microbial succession. *S. cerevisiae* reached peak abundance during mid-fermentation (days 8–16), corresponding with efficient ethanol production, while *Geotrichum silvicola* became dominant in the later stage, though its special function remains to be clarified. Moreover, in newly established pits, non-*Saccharomyces* yeasts maintained longer dominance, whereas older pits showed earlier establishment of *S. cerevisiae* as the dominant species (Zhang et al., 2021).

Functionally, different yeast species exhibited pronounced metabolic differences. *S. cerevisiae* showed a strong capacity for synthesizing ethanol and medium-chain fatty acid esters, particularly ethyl caproate (Dong et al., 2024a). Qiu et al. reported that *S. cerevisiae* shows excellent tolerance to organic acids and high ethyl caproate capability (Qiu et al., 2024a). Subsequent co-cultivation of this strain with *Clostridium* spp. in strong-flavor Baijiu fermentation further boosted ethyl caproate yields (Qiu et al., 2024b; Qiu et al., 2025). *P. kudriavzevii* was noted for its strong capacity to produce acetic acid and ethyl acetate (Dong et al., 2024a). Both *K. bulderi* and *K. exigua* exhibited prominent roles in organic acid metabolism (You et al., 2021; Dong et al., 2024a). Duan et al. (2024) found that *C. lusitaniae* tolerates ethanol concentrations up to 15% and could synthesize multiple fruity esters such as ethyl palmitate, ethyl octanoate, ethyl elaidate, ethyl phenylacetate, and ethyl valerate.

### Yeasts in strong-flavor Daqu

Strong-flavor Daqu, also referred to as medium-temperature Daqu (typically prepared at 50–55 °C), serves as the saccharification and fermentation starter in strong-flavor Baijiu production. It is characterized by a complex microbial community structure and diverse ecological functions. The relatively high temperature during preparation, open production environment, and variation in raw materials collectively shape a unique yeast community, laying the foundation for flavor precursor formation and a stable microecological system during subsequent fermentation (Ren et al., 2024). In recent years, researchers have employed high-throughput sequencing and culture-based techniques to investigate the community composition, succession dynamics, and functional traits of yeasts in strong-flavor Daqu.

*S. cerevisiae* is one of the most frequently detected dominant yeast in strong-flavor Daqu, consistently found in a wide range of samples (Pu et al., 2021; Yan et al., 2019a; Gou et al., 2015). In addition, *P. kudriavzevii* is another prevalent species, with its relative abundance reported to exceed 40% in certain Daqu samples (Yang et al., 2018). Other commonly occurring yeasts include *W. anomalus*, *I. orientalis*, *P. fermentans*, *Geotrichum candidum*, *Saccharomycopsis fibuligera*, *Yarrowia lipolytica*, *H. uvarum*, and *Kazachstania barnettii*, which together form the core yeast diversity structure of strong-flavor Daqu (Table 1) (Pu et al., 2021; Ren et al., 2024; Yan et al., 2019b; Gou et al., 2015).

During Daqu preparation and storage, yeast communities undergo marked dynamic changes. Yan et al. (2019b) systematically investigated yeast succession in strong-flavor Daqu, revealing that *S. cerevisiae* proliferated rapidly in the early phase, while *P. kudriavzevii* and *W. anomalus* became dominant at later stages. Yang et al. (2018) reported that *P. kudriavzevii* peaked during the heating phase, accounting for 43.2% of the total fungal abundance, indicating its strong thermotolerance and high-sugar adaptation. Ren et al. (2024) observed a gradual decline in the abundance of *W. anomalus* during storage, suggesting its primary role occurs in fresh Daqu, where it contributes to ester synthesis and enzyme activity.

Functionally, yeasts in strong-flavor Daqu not only facilitate ethanol production but also directly affect the production of flavor compounds and enzymatic activities essential to fermentation. Li et al. (2020) demonstrated that fortified inoculation with *S. cerevisiae*, *Hyphopichia burtonii*, and *C. lusitaniae* significantly enhanced the production of volatile compounds such as ethyl caproate and ethyl acetate. Gou et al. (2015) reported that *S. fibuligera* exhibited high amylase and protease activities, playing a crucial role in saccharification and flavor precursor accumulation. Pu et al. (2021) isolated 11 yeast strains from strong-flavor Daqu and assessed their capacities for sugar-alcohol conversion, ester biosynthesis, and environmental adaptability, highlighting the strong ester-producing potential of *Y. lipolytica* and *W. anomalus*. Liu S. et al. (2023) further proposed that the yeasts inoculation source from environmental or mature Daqu could substantially reshape the yeast community structure in newly prepared Daqu, providing a theoretical support for targeted microbial control strategies.

### Yeasts in sauce-flavor Baijiu

Sauce-flavor Baijiu, characterized by its sophisticated production techniques and extended manufacturing period, is distinguished by its unique “three-high” processes: high-temperature Daqu preparation, high-temperature stacking fermentation, and high-temperature distillation. Notable exemplars of this liquor category comprise the prestigious Moutai and Langjiu brands. The production approach involves complex procedures, extended fermentation cycles, and a highly diverse microecosystem. The fermentation processes are typically divided into two stages: stacking fermentation and pit fermentation, overall lasting about 37 days and repeated across eight fermentation rounds and seven liquor withdrawals. Throughout the entire cycle, a wide range of microbes are involved, among which yeasts play crucial roles in ethanol production, flavor compound biosynthesis, and ecological balance regulation (Wang, 2022).

*S. cerevisiae* is the most frequently detected dominant yeast species in sauce-flavor Baijiu fermentation, exhibiting strong sugar-fermenting ability and high ethanol tolerance (Chen et al., 2025; Meng et al., 2015; Wu et al., 2012). In addition, *P. kudriavzevii*, *I. orientalis*, *W. anomalus*, and *S. fibuligera* are commonly present (Table 1), with *P. kudriavzevii* displaying high metabolic activity and adaptability across various fermentation rounds and regions (Song et al., 2017; Li et al., 2022). Moreover, thermotolerant and stress-resistant yeasts such as *Z. bailii*, *Candida humilis*, and *K. exigua* demonstrate strong ecological adaptability (Table 1), remaining viable and metabolically active under the high-temperature and high-osmotic conditions of the stacking environment (Wu et al., 2012; Sun et al., 2012; Song et al., 2020).

During the eight rounds of alternating stacking and pit fermentation, the microbial community undergoes dramatic succession in response to time and environmental shifts. Chen et al. (2025) observed that during the first round of stacking fermentation, the initial yeast community was dominated by environmental species such as *S. fibuligera* and *T. delbrueckii*, which was subsequently replaced by *S. cerevisiae* and *P. kudriavzevii* as fermentation progressed. Hao et al. (2021) tracked the early fermentation phase using high-throughput sequencing and identified temperature and acidity as the key environmental drivers of yeast succession. Under conditions of elevated temperature and low water activity, *Z. bailii* and *H. uvarum* showed strong environmental resilience and were positively associated with the accumulation of volatile flavor compounds.

Yeasts are major contributors to the biosynthesis of key flavor compounds in sauce-flavor Baijiu, including higher alcohols, organic acids, and esters. Wu et al. (2015) reported that regulating the saccharification rates could significantly affect the aroma formation pathway of *S. cerevisiae*, improving the efficiency of ester metabolism. Meng et al. (2015) further showed that co-culturing *S. cerevisiae* with *Bacillus licheniformis* significantly increased the yeild of ethyl caproate and ethyl lactate, highlighting the potential metabolic synergy between yeast and bacteria. Moreover, yeasts such as *Pichia manshurica*, *Debaryomyces hansenii*, and *T. delbrueckii* exhibited strong abilities to produce flavor precursors, and their consistent presence across multiple fermentation rounds suggests their potential as functional strains for improving Baijiu quality (Sun et al., 2012; Chen et al., 2025).

The origins of yeasts in sauce-flavor Baijiu showed distinct environmental and regional specificity. Zhou et al. (2024) noted that airborne microbes are important external sources of yeasts in both surface Daqu and pit fermentation environments. Species such as *I. orientalis* and *W. anomalus* were continuously detected in workshop air, suggesting their long-term persistence in the brewing environment through a “back-pit accumulation” mechanism. Furthermore, some yeasts, including *K. exigua* and *Candida krusei*, were enriched in pit-bottom mud and residual raw materials, forming “secondary starter communities” that laid the microbial foundation for next fermentation round (Wang et al., 2016; Wu et al., 2013).

### Yeasts in sauce-flavor Daqu

Sauce-flavor Daqu, also known as high-temperature Daqu, is prepared under the highest temperature conditions among traditional starter types, with peak fermentation temperatures reaching 60–65 °C and high-temperature stages lasting up to 48 h or more. These extreme conditions impose intense selective pressure on the microbial community, allowing only thermotolerant, acid-tolerant, and osmotolerant yeasts to survive and function effectively in this system.

*S. cerevisiae* is widely distributed and plays a vital role in sauce-flavor Daqu due to its thermotolerance and efficient glycolytic capacity. It is particularly active during the temperature rise period, where it rapidly consumes reducing sugar and proliferation, making it one of the core functional yeasts in this system (Liu et al., 2012; Wang et al., 2008; Zhu et al., 2023). In addition to *S. cerevisiae*, a variety of non-*Saccharomyces* species (Table 1), including *P. kudriavzevii*, *W. anomalus*, *S. fibuligera*, *H. uvarum*, *Candida stellata*, *D. hansenii*, and *Z. bailii*, are also widespread and demonstrate strong ecological adaptability, contributing significantly to the yeast diversity of sauce-flavor Daqu (Fan et al., 2024; Yi et al., 2019; Zhu et al., 2023).

The production of Daqu involves multiple stages, including sampling, stacking, heating, turning, and storage, during which microbial communities undergo dynamic succession. In the early stages, environmental yeasts such as *P. kudriavzevii* and *S. fibuligera* dominate; as the temperature rises, thermophilic species like *S. cerevisiae* and *W. anomalus* become increasingly abundant (Fan et al., 2024; Shi et al., 2022). Shi et al. (2022) further observed that the high-temperature core regions are mainly enriched with thermotolerant *S. cerevisiae* and rare yeasts species such as *Candida allociferrii*, while the cooler marginal regions harbor more environmental yeasts like *H. uvarum*, highlighting pronounced spatial heterogeneity. Comparative studies of mature Daqu types (white, yellow, and black) have shown that *Pichia* and *Candida* were dominant genera, with the highest yeast diversity observed in yellow Daqu, possibly due to its moderate temperature and humidity that favor yeast growth (Deng et al., 2020; Dong et al., 2024b).

Yeasts in sauce-flavor Daqu not only need to withstand high temperatures but also execute critical metabolic functions under these extreme conditions. Wu et al. (2023) performed transcriptomic analyses and revealed that under heat stress, *S. cerevisiae* and *S. fibuligera* showed significantly upregulate genes involved in heat shock protein synthesis and membrane stability, reflecting specialized thermotolerance strategies. *S. fibuligera* also exhibits strong hydrolytic enzyme activity, particularly amylase and protease, and is regarded as a key functional yeast during high-temperature saccharification (Zhu et al., 2023). *P. kudriavzevii* and *W. anomalus* possess strong aroma-producing capabilities, contributing to the formation of higher alcohols and esters, thus playing essential roles in flavor development. *Z. bailii* showed distinct gene expression related to amino acid metabolism, pyruvate breakdown, and fatty acid biosynthesis, indicating its potential metabolic contribution to the formation of Daqu’s flavor precursors (Yi et al., 2019).

The yeast community in Daqu is shaped not only by the wheat-based raw materials but also by the surrounding processing environment. Cai et al. (2021) found that yeast composition is influenced by multiple environmental factors, including fermentation temperature, ventilation, turning frequency, and room-level microecology. Fan et al. (2024) emphasized that air, tools, and turning personnel are important sources of yeast introduction. They recommended applying environmental microbial control techniques, such as air filtration and heated flooring, to promote the stable colonization of target yeasts in the production environment.

### Yeasts in light-flavor Baijiu

Light-flavor Baijiu is characterized by its clean and pure aroma, mild sweetness, smooth mouthfeel, and refreshing aftertaste. It is one of the earliest Baijiu aroma types to achieve industrialization and standardized production in China, with representative products including Fenjiu (Shanxi) and Hengshui Laobaigan (Hebei). This style of Baijiu typically adopts ground-jar fermentation, characterized by relatively short fermentation periods (28–45 days), with the microbial community structure primarily shaped by Daqu and natural environmental inoculation.

*S. cerevisiae* is the predominant yeast species in light-flavor Baijiu fermentation and is consistently present throughout all fermentation stages, playing a key role in ethanol production and primary flavor development (Cai et al., 2022; Chen et al., 2022; Wang and Xu, 2019). Additionally, various non-*Saccharomyces* yeasts such as *P. kudriavzevii*, *W. anomalus*, *Candida tropicalis*, *I. orientalis*, *K. exigua*, and *T. delbrueckii* have been reported (Table 1). These yeasts exhibit distinct ecological adaptability and metabolic activities at different fermentation stages and contribute significantly to the formation of characteristic light-flavor Baijiu aromas (Li et al., 2011; Huang et al., 2020; Xue et al., 2023).

The ground-jar fermentation system, with its periodic cycles and stratified feeding processes, led to a clear stage-dependent variation in yeast community composition. Wang and Xu (2019) found that *Pichia* species dominated in the early stages of fermentation, while *S. cerevisiae* gradually became dominant as the environment acidified. Cai et al. (2022) further revealed structural differences between yeast communities in the upper and lower layers of the fermented grains: the lower layer was enriched in acid- and ethanol-tolerant strains such as *S. cerevisiae*, whereas the upper layer harbored aroma-producing yeasts like *Kazachstania humilis* and *Hanseniaspora osmophila*. The succession of yeast communities is mainly driven by environmental factors such as temperature, acidity, and moisture (Kang et al., 2022).

Functionally, different yeast species exhibit significant division of labor during light-flavor Baijiu fermentation. Chen et al. (2022) isolated multiple strains of *P. kudriavzevii*, *W. anomalus*, and *S. cerevisiae* from fermented grains and found distinct differences in their capacities to synthesize alcohols, esters, and acids. Their synergistic interactions were critical for developing the typical aroma of light-flavor Baijiu. Furthermore, *I. orientalis* and *Cyberlindnera meyerae* were highly active in organic acid metabolism (especially lactic acid and pyruvate), suggesting a dual role in ecological modulation and flavor ester biosynthesis, such as ethyl acetate and ethyl lactate (Huang et al., 2020; Luo et al., 2023). Yeast species like as *D. hansenii*, *T. delbrueckii*, and *Z. bailii* observed across multiple fermentation cycles, participating in the production of higher alcohols and thereby enhancing aroma complexity of light-flavor Baijiu (Wang and Xu, 2019).

From a microecological perspective, yeasts in light-flavor Baijiu often form mutualistic relationships with LAB. Chen et al. (2022) found that *S. cerevisiae* inhibited undesirable microbial growth and created a favorable environment for LAB development. Meanwhile, organic acids produced by *P. kudriavzevii* and *W. anomalus* helped regulate the microbial balance and enhanced system stability. Xue et al. (2023) conducted metagenomic analyses and showed that yeasts are highly active in pathways related to amino acid degradation, fatty acid biosynthesis, and glycolysis, and their metabolic cooperation with LAB further promotes flavor compound accumulation.

### Yeasts in light-flavor Daqu

Light-flavor Daqu, also known as low-temperature Daqu, is typically prepared at 45–50 °C. Unlike the medium-temperature Daqu used in strong-flavor Baijiu or the high-temperature Daqu in sauce-flavor Baijiu, low-temperature Daqu features moderate temperature and has a relatively short fermentation cycle of 25–30 days. The main raw material is wheat, and the Daqu blocks are produced by natural stacking fermentation in fermentation rooms, allowing yeast communities to undergo succession in a relatively mild environment.

*S. cerevisiae* is widely present in light-flavor Daqu and is generally considered a dominant species, primarily responsible for sugar metabolism, ethanol production, and the formation of primary flavor compounds (Zheng et al., 2012; Hu et al., 2021). Non-*Saccharomyces* yeasts such as *P. kudriavzevii*, *W. anomalus*, *Candida guilliermondii*, *S. fibuligera*, *I. orientalis*, and *Meyerozyma guilliermondii* also play key roles during Daqu fermentation (Table 1), particularly in starch hydrolysis, organic acid metabolism, and flavor compound biosynthesis (Li R. Y. et al., 2018; Shi et al., 2009; Xiang et al., 2023). Notably, species like *M. guilliermondii* has only been found in some light-flavor Daqu samples, which show strong regional specificity and suggesting that yeast community composition is strongly affected by the geographic environment of Daqu production (Xiang et al., 2023).

The composition of microbial communities in light-flavor Daqu is influenced by multiple factors including raw materials, water quality, climate, production technology, and environmental microecology. Xiang et al. (2023) compared Daqu samples from Taiyuan (Shanxi) and Suizhou (Hubei) and found that yeast community diversity differed significantly between regions. *S. cerevisiae* and *P. kudriavzevii* dominated in samples from Shanxi, while *S. fibuligera* and *K. humilis* were more abundant in samples from Hubei, indicating that ecological and process differences jointly shape the yeast community. Zheng et al. (2014) also reported that in the low-temperature Daqu used for Fenjiu, yeast communities were more easily established and stabilized in high-moisture, low-temperature environments, and that flipping frequency and drying procedures during Daqu making significantly affected community succession speed and dominance patterns.

In addition to ethanol production, yeasts in light-flavor Daqu also actively participate in saccharification, precursor transformation, and synthesis of characteristic flavor compounds. *S. fibuligera* has strong amylase and protease activities and is recognized as a typical saccharifying yeast (Hu et al., 2021). *W. anomalus* and *P. kudriavzevii* show outstanding capabilities in ester and alcohol biosynthesis, particularly in producing ethyl acetate and ethyl lactate (Li R. Y. et al., 2018; Zheng et al., 2023). Transcriptomic analysis by Zheng et al. (2023) revealed that *S. cerevisiae* and *D. hansenii* upregulated genes related to glycolysis and fatty acid biosynthesis during the high-sugar phase of Daqu fermentation, suggesting their roles in sugar and lipid metabolism. *C. guilliermondii* and *I. orientalis* were involved in the synthesis of pyrroles, furans, and other secondary metabolites, contributing to the aromatic complexity of light-flavor Baijiu (Shi et al., 2009).

From a microecological perspective, yeasts in light-flavor Daqu interact closely with molds (e.g., *Aspergillus oryzae*) and bacteria (e.g., *Lactobacillus plantarum*). Pan et al. (2022) used metatranscriptomics to show that yeasts such as *W. anomalus* and *Cyberlindnera jadinii* upregulate metabolic pathways of some organic acids to enhance acid tolerance and stabilize LAB within Daqu blocks. Luo et al. (2022) proposed a “yeast–LAB co-fermentation model,” suggesting that *S. cerevisiae* regulates ethanol and higher alcohol levels to support LAB growth, while organic acids produced by LAB, in turn, promote ester-producing functionality in yeasts.

## Evolution, phenotypic and metabolic characteristics

Yeasts play a dominant role in Baijiu brewing due to their unique metabolic capabilities and environmental adaptability, particularly in ethanol production and the biosynthesis of various flavor compounds. The evolution and differences in phenotypic and metabolic traits directly influence Baijiu’s flavor profile, product stability, and process controllability. In recent years, with the rapid development of omics technologies, researches on yeast metabolite profiles, environmental adaptation mechanisms, and key functional genes has deepened significantly.

### Evolutionary origin and domestication of yeasts in Baijiu ecosystems

Yeasts in Baijiu fermentation have undergone remarkable evolutionary divergence under the unique conditions of traditional solid-state fermentation, including high temperatures, acidic environments, ethanol accumulation, and close microbial interactions. Among these, *Saccharomyces cerevisiae* is widely recognized as the core ethanol-producing species and has experienced lineage-specific domestication shaped by both natural selection and long-term human practices.

Multiple genomic and ecological studies support East Asia, particularly China, as the center of origin and domestication for *S. cerevisiae*. Duan et al. (2018) demonstrated that Chinese strains diverged into distinct clades associated with solid- and liquid-state fermentations, likely from admixed wild ancestors. Lee et al. (2022) further revealed deeply divergent pre-domesticated lineages in Taiwan forests, suggesting a complex history of natural diversity prior to fermentation-driven selection. Large-scale population analyses by Loegler et al. (2024) uncovered four superclades of *S. cerevisiae*, Wine, Beer, Asian Fermentation, and Wild, with Baijiu-related strains forming a polyploid, heterozygous cluster within the Asian Fermentation clade. These populations exhibit adaptive traits including heat resistance, acid tolerance, and flavor compound biosynthesis. Peris et al. (2023) expanded this framework by illustrating macroevolutionary divergence across the *Saccharomyces* genus, driven by geography and fermentation ecology. In addition to *S. cerevisiae*, non-*Saccharomyces* yeasts such as *Wickerhamomyces*, *Pichia*, *Zygosaccharomyces*, and newly described taxa, play important roles in Baijiu production. Hu et al. (2025) emphasized the phylogenetic novelty and ecological plasticity of yeasts isolated from ethnic minority fermented foods, including environments analogous to Daqu. This suggests that non-conventional yeasts may also undergo domestication-like selection within high-stress fermentation niches. Sampaio and Pontes (2025) proposed that yeast domestication is a continuum shaped by ecological constraints, bottlenecks, and gene flow. In Baijiu, this process manifests through metabolic specialization, genome plasticity, and population differentiation linked to substrate type, fermentation style, and cultural practices. Together, these findings highlight Baijiu fermentation as both a cradle and crucible of yeast evolution. It preserves wild diversity while promoting adaptive innovation, offering a valuable model for studying microbial domestication in traditional food systems.

### Metabolite profiles and flavor contributions of yeasts

A key metabolic feature of yeasts in Baijiu fermentation is their capacity to synthesize aroma compounds. *S. cerevisiae* produces ethanol, ethyl acetate, ethyl lactate, higher alcohols, and short-chain fatty acids via glycolysis and fatty acid metabolism pathways (Zhang et al., 2024; Yang et al., 2022). For example, the strain *S. cerevisiae* strain NJ002, isolated from high-temperature Daqu, exhibited strong ester-producing capacity, efficiently generating ethyl acetate and ethyl caproate (Wang Y. et al., 2023). Dong et al. isolated *S. cerevisiae* FJ1-2 from fermented grains of strong-flavor Baijiu, which produced medium-chain fatty acids and esters, reaching 10.5 mg/L of ethyl caproate in a sorghum juice medium (Dong et al., 2024a).

Non-*Saccharomyces* yeasts also play crucial roles in flavor formation. Strains such as *P. kudriavzevii*, *Pichia anomala*, *W. anomalus*, *Candida parapsilosis*, *Z. bailii*, *Kazachstania bulderi*, *S. fibuligera*, and *Schizosaccharomyces pombe* contribute to diverse aroma profiles. For example, *P. kudriavzevii* FJ1-3, isolated from strong-flavor Baijiu, demonstrated high tolerance to 45 °C and strong acetic acid and ethyl acetate production (Dong et al., 2024a). *P. anomala* was identified as a high ethyl acetate-producing strain, with optimized fermentation yielding 4.812 g/L of ethyl acetate (Chen et al., 2011). Wang et al. (2020) reported that adding *W. anomalus* Y3604 in Baijiu fermentation significantly increased ethyl acetate and ethyl caproate levels while reducing higher alcohol. *C. parapsilosis* exhibited strong biosynthesis ability for ethyl caproate and ethyl lactate, making it a promising functional strain (Wang et al., 2024). *Z. bailii* produced phenylethanol, γ-valerolactone, ethyl benzoate, and other aromatic compounds through multiple pathways, enriching the aroma complexity of Baijiu (Xu et al., 2017). *K. bulderi* displayed acid tolerance and modulated the yeast community to favor high ethyl caproate yields with low ethyl acetate production in strong-flavor Baijiu (Dong et al., 2024a). *S. fibuligera*, frequently found in Daqu, significantly increased ethanol and total ester levels when fortified application in Baijiu fermentation (Su et al., 2020).

### Environmental adaptability and stress-tolerant phenotypes of yeasts

In traditional solid-state fermentation systems, yeasts face stressors such as high temperature, acidity, and ethanol concentration. Their ability to survive and function under these conditions reflects diverse stress-tolerant phenotypes. Lu et al. isolated a strain of *S. cerevisiae* from the typical Maotai production environment in sauce-flavor Baijiu and named it MT1. This strain showed remarkable stress tolerance under conditions of pH 2.0, 16% ethanol, and temperatures up to 44 °C (Lu et al., 2015). *P. kudriavzevii*, a representative acetic acid-tolerant yeast, maintained intracellular homeostasis through the upregulation of membrane transporter genes, while also exhibiting strong ethanol-producing capacity under acetic acid condition of pH 2.73 (Wang N. et al., 2023). *Z. bailii* is particularly resistant to both lactic acid and high ethanol concentrations. Wei et al. (2023) found that *Z. bailii* alleviated acid stress by activating intracellular proline and serine synthesis pathways, and maintained mitochondrial function and respiratory chain activity, making it an excellent functional strain for environments with multiple stresses. *S. pombe* tolerated 10 g/L acetic acid and responded to stress by upregulating genes such as *ACCAT1*, *HMGCS1*, and *HMGCR1*, which were involved in acetate metabolism (Song et al., 2019). To better compare stress tolerance traits across ecosystems, we summarized key phenotypic characteristics of representative non-*Saccharomyces* yeasts in Table 2. This table highlights species-specific acid and thermal tolerances, their ecological origin, and dominant metabolic functions. The compilation demonstrates how these yeasts adapt to distinct fermentation conditions in strong-flavor, sauce-flavor, and light-flavor Baijiu production, further revealing their complementary ecological roles and potential applications.

**Table 2:** Functional traits of representative non-*Saccharomyces* yeasts in Baijiu ecosystems.

Species	Ecosystem	Acid tolerance (pH)	Thermotolerance (°C)	Key functional traits	References
*Pichia kudriavzevii*	Strong-flavor Baijiu yellow-water	pH < 3.0	Thermotolerant (~45 °C)	Acetic acid resistance (up to 12 g/L)	Wang N. et al. (2023)
*Zygosaccharomyces bailii*	Sauce-flavor Baijiu	pH ~ 3.2	Moderate	Lacetic acid resistance (up to 50 g/L)	Wei et al. (2023)
*Schizosaccharomyces pombe*	Sauce-flavor Baijiu	pH < 3.0	Moderate	Acetic acid resistance (up to 10 g/L)	Song et al. (2020)
*Kazachstania exigua*	Strong-flavor Baijiu	pH ~ 3.5	Moderate	Acid modulation	You et al. (2021)
*Wickerhamomyces anomalus*	Sauce-flavor Daqu	pH < 4.0	Thermotolerant (~45 °C)	Ester formation; Antibacterial activity	Kang et al. (2024), Li et al. (2024)
*Issatchenkia orientalis*	Strong-flavor pit mud	pH < 4.0	Moderate	Ester production	Wang and Xu (2019)
*Saccharomycopsis fibuligera*	Light-flavor Daqu	Neutral	Moderate	Amylase; Starch degradation	Su et al. (2020), Zhang et al. (2024)
*Hanseniaspora uvarum*	Sauce-flavor Daqu	Neutral	Low	Aroma enhancement (fruity)	Li et al. (2022)
*Torulaspora delbrueckii*	Various Daqu	Neutral	Moderate	Flavor enhancement; mixed fermentation	Li et al. (2024)
*Yarrowia lipolytica*	Strong-flavor Daqu	Neutral	Moderate	Fatty acid synthesis	Pu et al. (2021)

### Carbohydrate, amino acid, and lipid metabolism of yeasts

Yeasts differ in substrate utilization and metabolic flux. *S. fibuligera* exhibits potent saccharolytic and proteolytic capabilities, facilitating the hydrolysis of starch and proteins into fermentable sugars and amino acids. This metabolic activity significantly enhances the substrate utilization efficiency of Daqu during fermentation (Zhang et al., 2024). *I. orientalis* synthesizes distinctive sulfurous metabolites, including 3-methylthiopropanol, when cultured in sulfur-enriched amino acid substrates. This biochemical activity contributes significantly to the flavor complexity and aromatic diversity of Baijiu fermentation (Zha et al., 2017). Genomic and transcriptomic analysis of *S. cerevisiae* strain MT1 by Lu et al. (2015) revealed elevated expression of critical enzyme-encoding genes, including *β*-galactosidase, fructokinase, and lipase. This genetic profile enables the strain to efficiently utilize diverse carbon sources (e.g., glucose, fructose, lactose, and lipids), thereby facilitating vigorous alcoholic fermentation in complex substrate environments.

### Functional genes and metabolic pathway analysis of yeasts

Advances in yeast genomics and transcriptomics have progressively revealed critical metabolic pathways and regulatory genes involved in Baijiu brewing. In *S. cerevisiae*, the alcohol acetyltransferase-encoding genes *ATF1* and *ATF2* demonstrate temperature- and pH-dependent expression patterns, directly modulating ethyl acetate production and consequently influencing ester profiles and yields (Yang et al., 2022). Genomic studies of *Z. bailii* have identified multiple gene clusters associated with the phenylethanol biosynthesis pathway, indicating their potential for precise modulation of this key aromatic compound (Xu et al., 2017). Thiol metabolism studies revealed that *S. cerevisiae* genes STR3 (cysteine β-lyase) and CYS3 (cystathionine β-synthase) coordinately regulate 2-furfurylthiol (2-FFT) biosynthesis - a potent aroma-impacting compound (Zha et al., 2017). These genetic elements present valuable targets for flavor engineering in Baijiu production.

### Advances in artificial selection and functional optimization of yeasts

Recent studies have increasingly focused on isolating and functionally enhancing yeast strains from traditional fermentation systems to improve aroma production, stress tolerance, and substrate utilization efficiency. For instance, Zhang et al. (2024) screened a *S. cerevisiae* mutant with high 3-methylthiopropanol production and significantly enhanced its yield through optimized amino acid substrate availability and pH regulation. Similarly, Li P. et al. (2017) demonstrated that co-inoculating *W. anomalus* and *S. fibuligera* in Daqu systems could synergistically elevate esterase and amylase activities, providing a novel approach for constructing functionally tailored microbial consortia. In another breakthrough, Zhao et al. isolated *Candida* sp. ZY002, an ethyl caproate-producing strain, achieving a titer of 170.56 mg/L through fermentation optimization. When applied in fortified Daqu, this strain significantly increased ethyl caproate levels in fermented grains (Zhao et al., 2024). Further advancing this field, Qiu et al. (2024a) identified *S. cerevisiae* YS219, a high-performance strain capable of producing 61.11 g/L ethyl caproate. Implementation of YS219 in fermentation boosted total ester and ethanol contents by 44.3 and 188.4%, respectively, with ethyl caproate reaching 214.367 mg/kg, a 34 mg/kg increase over controls. These studies collectively demonstrate the tremendous potential of targeted yeast selection and metabolic optimization for enhancing Baijiu quality and production efficiency. These advances in strain selection and functional enhancement not only demonstrate the applied potential of phenotypic and metabolic insights, but also provide essential groundwork for subsequent genetic engineering strategies. The following sections will further explore the discovery of novel yeast species and the molecular tools used to enhance their functionality in Baijiu fermentation.

## Discovery and characterization of novel yeast species

The application of advanced microbial taxonomy and molecular biology techniques has led to the identification of numerous novel yeast species from Baijiu fermentation environments. These discoveries significantly expand our understanding of microbial diversity in traditional fermentation systems while providing new insights into their functional roles in flavor development, ecological adaptation, and potential biotechnological applications.

Lei et al. (2022) isolated *Moniliella aeria* sp. nov. from the brewing workshop air at Wuliangye (Yibin, Sichuan Province), this black yeast demonstrates robust metabolic capabilities, efficiently utilizing sucrose, xylose, and mannose. Its thermotolerance (30–37 °C), moderate ethanol resistance, and adaptability to high-sugar, high-humidity conditions suggest long-term survival potential in fermentation environments and possible ecological contributions. Wei et al. (2024) reported the isolation of *Starmerella fangiana* f.a. sp. nov. from Daqu production regions in Sichuan. This species exhibits broad substrate utilization (glucose, fructose, mannose) and produces flavor-active esters and medium-chain fatty acid esters. Its remarkable tolerance to high temperatures and osmotic stress highlights its potential as a functional starter culture. Zhen et al. (2025) isolated a novel species *Ascoidea xinghuacunensis* sp. nov. from the historical brewing site of Xinghuacun in Shanxi Province. Here, the first *Ascoidea* species was reported from Chinese Baijiu environments (Xinghuacun, Shanxi). This strain thrives in slightly acidic to neutral pH and may contribute to aldehydes, ketones, and carboxylic acid formation. Its phylogenetic uniqueness and aroma-modulating potential make it valuable for both evolutionary studies and industrial applications.

## Genetic engineering and functional enhancement of yeasts

With the development of genetic engineering and synthetic biology, researchers have increasingly explored genetic modification strategies to enhance yeast functions during Baijiu fermentation. These efforts aim to improve aroma productivity, stress resistance, and the biosynthetic efficiency of target metabolites. Such studies not only enrich the applications of brewing microbes but also provide powerful tools for precise flavor modulation in Baijiu production.

### Regulation of ester compound synthesis

Ester compounds, including ethyl acetate, ethyl lactate, ethyl butyrate, and ethyl caproate are critical flavor compounds in Baijiu, contributing significantly to Baijiu flavor profiles. Recent advances in metabolic engineering have enabled precise regulation of their biosynthesis. Chen et al. (2014) demonstrated that overexpression of the *EHT1* combined with knockout of *FAA1* significantly increased the yield of ethyl caproate by redirecting acyl-CoA flux. Further refinement in ester synthesis control was achieved by Cui et al. (2018), who developed a *PGK1* promoter library for tunable expression of key enzymes, allowing quantitative adjustment of ethyl acetate levels. For broader ester profile modulation, Li et al. (2017a, 2017b) implemented a modular strategy involving *BAT2* deletion coupled with overexpression of alcohol acetyltransferase genes (*ATF1* and *ATF2*), enabling directed biosynthesis of diverse esters to meet specific aroma requirements.

### Biosynthesis of non-traditional metabolites

Beyond traditional esters, metabolic engineering has facilitated the production of novel flavor-active compounds in Baijiu. Hu et al. successfully introduced a heterologous monoterpene pathway into *S. cerevisiae*, enabling *de novo* biosynthesis of D-limonene, which is a compound with both aromatic and potential health-promoting properties (Hu Z. et al., 2020). Similarly, Cui et al. (2020) engineered *S. cerevisiae* for enhanced synthesis of 2,3,5,6-tetramethylpyrazine (TMP), a neuroprotective compound that also contributes roasted and nutty flavor notes, through optimization of precursor supply and elimination of competing pathways.

### Carbon flux modulation for targeted flavor substances

Strategic manipulation of central carbon metabolism has proven effective for boosting specific flavor compounds. Li W. et al. (2018) optimized higher alcohol production by fine-tuning the expression of key pyruvate kinase (*PYK1*) and alcohol dehydrogenase (*ADH1*) in glycolysis, resulting in elevated levels of butanol and isoamyl alcohol. In parallel, Ren et al. (2020) improved ethyl lactate yield by disrupting competing pathways and introducing a heterologous lactate esterification module, thereby enhancing the fruity and acidic flavor characteristics of Baijiu.

### Integrated strain improvement strategies

The convergence of genome editing and classical breeding techniques has opened new avenues for yeast strain development. Sun et al. (2025) employed CRISPR/Cas9-mediated ploidy engineering to construct polyploid *S. cerevisiae* strains with enhanced ethyl acetate production and improved stress resistance. These strains not only exhibited superior ester synthesis capacity but also demonstrated remarkable tolerance to fermentation-associated stresses, including low pH and elevated temperature. Such integrated approaches highlight the potential of combining cutting-edge genetic tools with traditional microbiological methods to develop robust, high-performance starter cultures for Baijiu production.

## Conclusions and future perspectives

**Figure 2:**
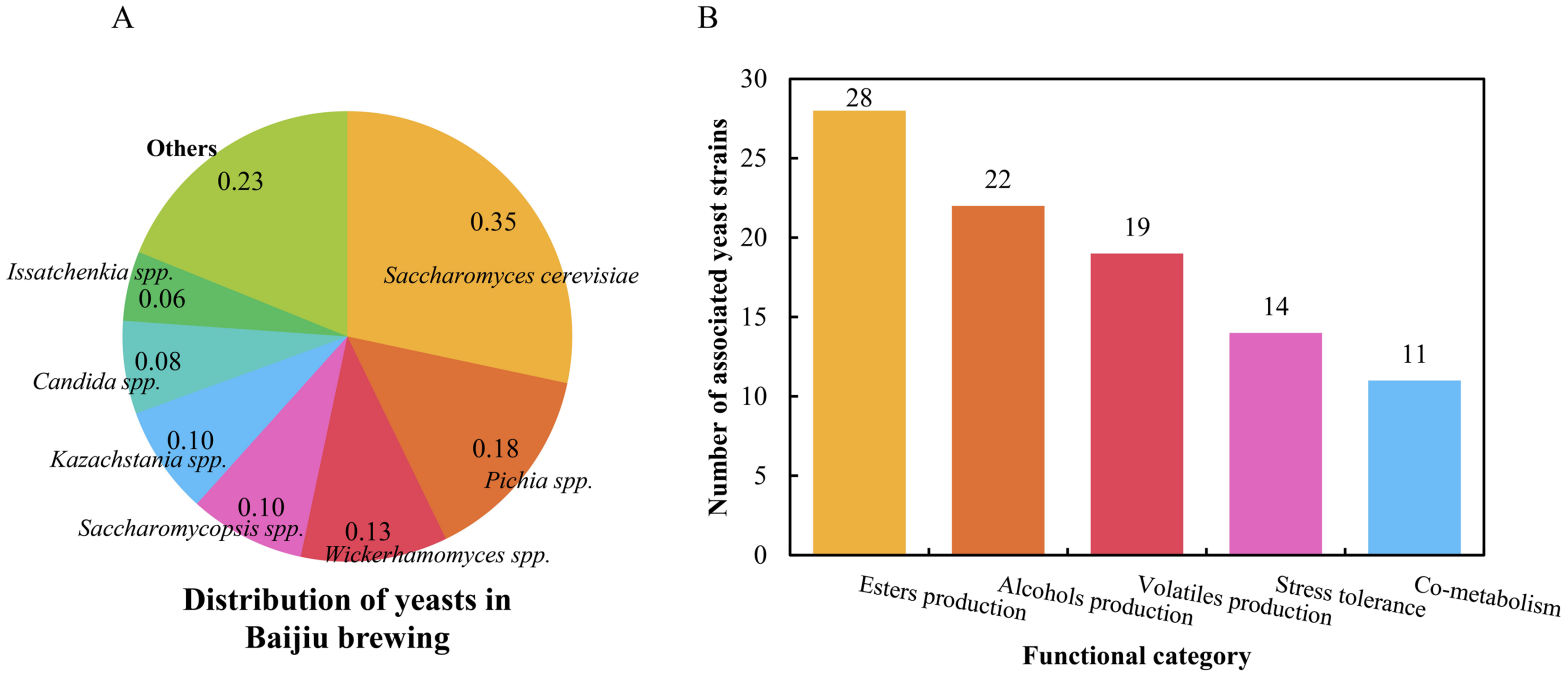
Distribution of yeasts in Baijiu brewing **(A)** and key functional classification of yeasts **(B)** in this review.

**Figure 3:**
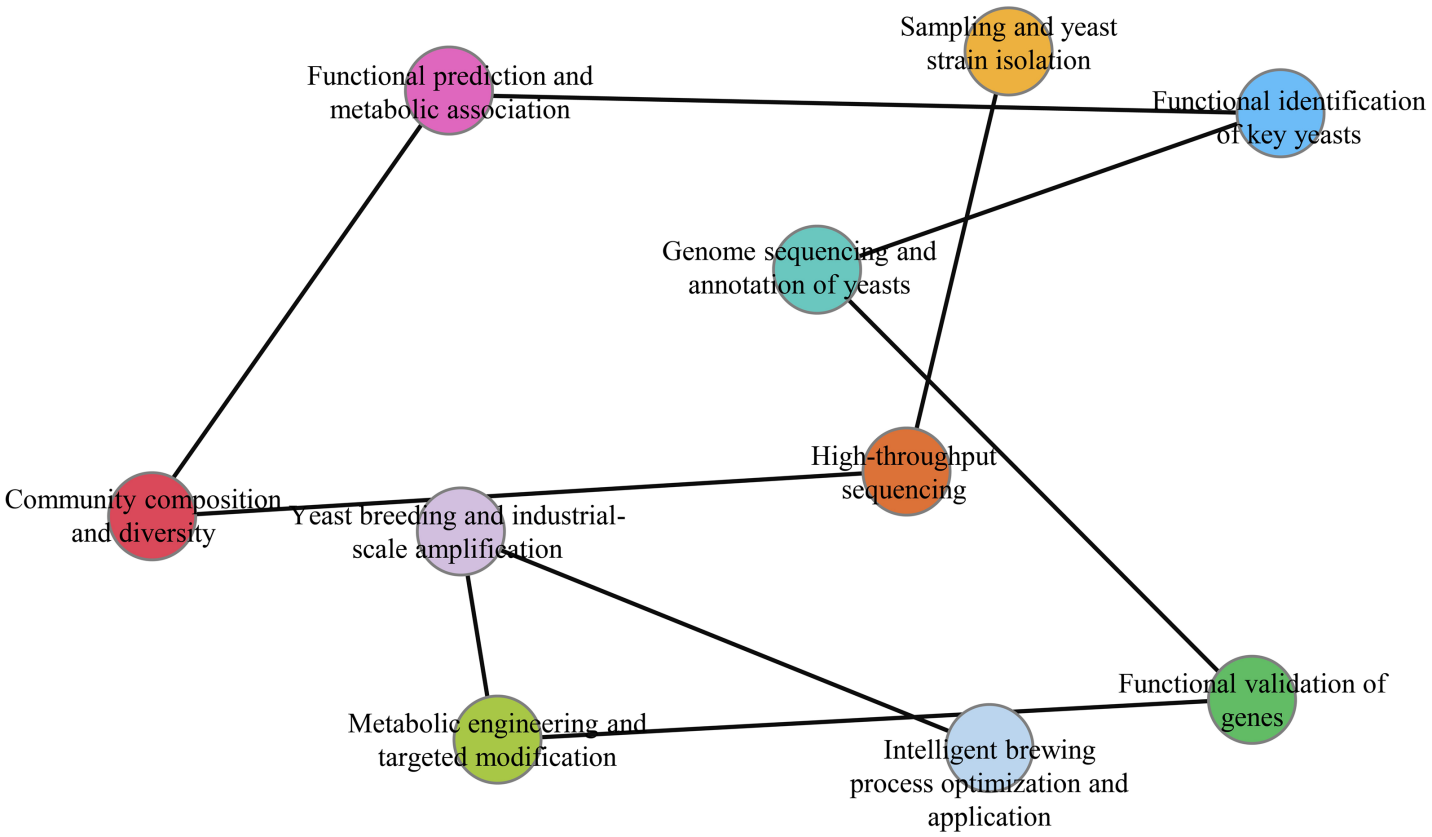
Research workflow and hotspots of yeasts in Baijiu brewing. Here, the main top 10 hotspots were showed as follow. (1) Sampling and yeast strain isolation, (2) high-throughput sequencing, (3) community composition and diversity, (4) functional prediction and metabolic association, (5) functional identification of key yeasts, (6) genome sequencing and annotation of yeasts, (7) functional validation of genes, (8) metabolic engineering and targeted modification, (9) yeast breeding and industrial-scale amplification, (10) intelligent brewing process optimization and application.

As a hallmark of traditional Chinese fermentation technology, Baijiu production relies on intricate microbial interactions to develop its characteristic aroma profiles. Yeasts serve as pivotal catalysts in this system, with *S. cerevisiae* remaining the core functional species (Figure 2A), while non-*Saccharomyces* yeasts (*Pichia* spp., *Wickerhamomyces* spp., *Saccharomycopsis* spp., *Kazachstania* spp., *Candida* spp., *Issatchenkia* spp. et al.) demonstrate expanding roles in ester/alcohol production, volatile biosynthesis, stress tolerance, and cross-species co-metabolism (Figure 2B). Our bibliometric analysis reveals ten key research hotspots (Figure 3) that collectively map the evolution from fundamental discovery to application: (1) Sampling and yeast strain isolation, (2) High-throughput sequencing, (3) Community composition and diversity, (4) Functional prediction and metabolic association, (5) Functional identification of key yeasts, (6) Genome sequencing and annotation of yeasts, (7) Functional validation of genes, (8) Metabolic engineering and targeted modification, (9) Yeast breeding and industrial-scale amplification, and (10) Intelligent brewing process optimization and application. This progression mirrors the field’s maturation from descriptive ecology to predictive systems biology. Future research priorities should address:

### Exploration, functional evaluation, and establishment of yeast repository

It is well recognized that current cultivation techniques limit our ability to culture only about 1–5% of microbial diversity. As a representative of extreme fermentation environments characterized by high acidity and ethanol concentrations, Baijiu brewing undoubtedly harbors a wealth of undiscovered microbial resources, particularly yeast strains. Systematic exploration of these resources, especially novel species and functionally unique yeasts, will significantly enrich both the diversity and functional capacity of available yeast collections, providing core strains for Baijiu production. With the continuous discovery of novel and functionally important yeast strains, there is an urgent need to establish dedicated preservation platforms and standardized evaluation systems. These should comprehensively assess key characteristics including temperature/sugar/acid/ethanol tolerance, ester/alcohol production profiles, aroma compound synthesis, and novel bioactive compound generation. Such efforts will facilitate the transition from resource discovery to practical application, while advancing consumer experiences from mere enjoyment to comfort and health-conscious consumption.

### Unlocking application potentials of yeast resources

Following the establishment of yeast repositories, it is essential to explore the broader application potential of Baijiu-derived yeast strains beyond conventional ethanol fermentation. Many strains, especially non-*Saccharomyces* yeasts such as *Rhodotorula*, *Wickerhamomyces*, *Pichia*, and *Zygosaccharomyces*, possess biosynthetic capabilities for producing high-value compounds. These include bulk fermentation chemicals (e.g., higher alcohols, organic acids), bioactive peptides, natural colorants (e.g., carotenoids), antioxidants, and other functional metabolites. This metabolic versatility makes them promising candidates for use in food preservation, functional foods, cosmetics, pharmaceuticals, and clean-label ingredient production. Future research should integrate phenotype screening with multi-omics analysis and bioprocess engineering to identify and optimize strains with desirable metabolic outputs. These efforts will not only expand the industrial utility of traditional fermentation yeasts but also align with the growing demand for sustainable, functional, and culturally-rooted microbial biomanufacturing.

### Advancing diversity characterization

Current understanding of yeast communities remains constrained by the limitations of amplicon sequencing, which typically provides genus-level resolution and relative abundance data. This approach hinders precise identification at species/subspecies/strain levels and prevents meaningful cross-sample comparisons of absolute microbial abundance, particularly critical in Baijiu’s high-acidity environment where rapid microbial succession occurs. Moreover, growing evidence indicates substantial intraspecific diversity within dominant yeast species such as *S. cerevisiae* and other non-*Saccharomyces*. Strains from different ecological or geographic origins often exhibit significant variation in ethanol production, stress tolerance, and metabolic outputs, reflecting lineage-specific adaptation and domestication. Recognizing and characterizing this strain-level diversity is crucial for understanding functional differentiation and fermentation performance in Baijiu ecosystems. Future research should employ quantitative amplicon sequencing integrated with culturomics, metagenomics, metatranscriptomics, and metabolomics to accurately determine absolute yeast populations and functional dynamics. Such multi-omics approaches will enable fine-scale resolution of “strain-function-niche” relationships and reveal succession patterns and key functional pathways throughout fermentation.

### Integrated engineering strategies for strain improvement

Future yeast optimization must transcend traditional mutagenesis or single-gene modifications by developing synthetic biology platforms incorporating CRISPR/Cas systems, high-throughput multi-trait screening, systems metabolic engineering, and synthetic chromosome design. While these technologies enable precise metabolic flux control and systematic optimization for specific aroma profiles, practical application requires careful consideration. Engineered strains must demonstrate long-term adaptability and functional stability under natural fermentation conditions before industrial deployment, necessitating rigorous ecological validation alongside laboratory characterization.

### Toward systemic fermentation control

Comprehensive understanding requires integrating yeast studies with bacterial and fungal co-metabolism networks. Developing unified “community-metabolic network-flavor output” models through metabolic flux analysis (MFA), stable isotope tracing (13C), metabolomics, and quantitative sequencing will quantify critical pathways and microbial dynamics. Combining these with synthetic microbial consortia construction, microecological regulation strategies, and functional community evaluation will enable directed, intelligent, and health-conscious Baijiu production. However, the introduction of engineered strains or synthetic microbial communities into Baijiu fermentation, deeply rooted in tradition, faces considerable challenges. Traditional Baijiu is not only a product of microbial metabolism but also a cultural artifact shaped by inherited techniques, sensory expectations, and artisanal craftsmanship. Introducing exogenous functional strains may disrupt native micro-ecological balances and provoke resistance from producers and consumers alike. Therefore, any modernization strategy must strike a careful balance between functional optimization and cultural preservation. Future applications should prioritize minimal intervention strategies, such as precision modulation of native strains or guided succession using indigenous consortia, to ensure both technological advancement and heritage continuity. This systems-level approach will provide the microbiological foundation and technological support needed for intelligent brewing processes and global market expansion.

## References

[ref1] Cai W. Wang Y. Liu Z. Liu J. Zhong J. Hou Q.. (2022). Depth-depended quality comparison of light-flavor fermented grains from two fermentation rounds. Food Res. Int. 159:111587. doi: 10.1016/j.foodres.2022.111587, PMID: 35940787

[ref2] Cai W. Xue Y. Wang Y. Wang W. Shu N. Zhao H.. (2021). The fungal communities and flavor profiles in different types of high-temperature Daqu as revealed by high-throughput sequencing and electronic senses. Front. Microbiol. 12:784651. doi: 10.3389/fmicb.2021.784651, PMID: 34925290 PMC8674350

[ref3] Chen Z. P. Cao Q. Meng T. T. Shi W. Zhang X. J. Chai L. J.. (2025). Yeast community in the first-round fermentation of sauce-flavor baijiu: source, succession and metabolic function. Food Res. Int. 200:115466. doi: 10.1016/j.foodres.2024.115466, PMID: 39779106

[ref4] Chen Y. Li F. Guo J. Liu G. Guo X. Xiao D. (2014). Enhanced ethyl caproate production of Chinese liquor yeast by overexpressing EHT1 with deleted FAA1. J. Ind. Microbiol. Biotechnol. 41, 563–572. doi: 10.1007/s10295-013-1390-3, PMID: 24370880

[ref5] Chen M. Li R. Peng N. Zhen D. Fang S. (2011). Screening of higher yield of ethyl acetate yeast and researching on ester-producing conditions. Afr. J. Microbiol. Res. 5, 1383–1388. doi: 10.5897/ajmr10.420

[ref6] Chen C. Xiong Y. Xie Y. Zhang H. Jiang K. Pang X.-N.. (2022). Metabolic characteristics of lactic acid bacteria and interaction with yeast isolated from light-flavor baijiu fermentation. Food Biosci. 50:102102. doi: 10.1016/j.fbio.2022.102102

[ref7] Cui D. Y. Wei Y. N. Lin L. C. Chen S. J. Feng P. P. Xiao D. G.. (2020). Increasing yield of 2,3,5,6-Tetramethylpyrazine in baijiu through *Saccharomyces cerevisiae* metabolic engineering. Front. Microbiol. 11:596306. doi: 10.3389/fmicb.2020.596306, PMID: 33324376 PMC7726194

[ref8] Cui D. Y. Zhang Y. Xu J. Zhang C. Y. Li W. Xiao D. G. (2018). PGK1 promoter library for the regulation of acetate Ester production in *Saccharomyces cerevisiae* during Chinese baijiu fermentation. J. Agric. Food Chem. 66, 7417–7427. doi: 10.1021/acs.jafc.8b02114, PMID: 29939025

[ref9] Deng L. Mao X. Liu D. Ning X. Q. Shen Y. Chen B.. (2020). Comparative analysis of physicochemical properties and microbial composition in high-temperature Daqu with different colors. Front. Microbiol. 11:588117. doi: 10.3389/fmicb.2020.588117, PMID: 33329462 PMC7732550

[ref10] Dong W. Yu X. Wang L. Zou M. Ma J. Liu J.. (2024b). Unveiling the microbiota of sauce-flavor Daqu and its relationships with flavors and color during maturation. Front. Microbiol. 15:1345772. doi: 10.3389/fmicb.2024.1345772, PMID: 38328433 PMC10848165

[ref11] Dong W. Zeng Y. Ma J. Cai K. Guo T. Tan G.. (2024a). Characteristics and functions of dominant yeasts together with their applications during strong-flavor baijiu brewing. Foods 13:2409. doi: 10.3390/foods13152409, PMID: 39123600 PMC11311647

[ref12] Duan Z. F. Han M. Y. Niu J. L. Zhao J. R. Li W. W. Zhu L. N.. (2024). Evolution of fermented grain yeast communities in strong-flavored baijiu and functional validation of yeasts that produce superior-flavored substances. J. Sci. Food Agric. 104, 5973–5981. doi: 10.1002/jsfa.13423, PMID: 38436499

[ref13] Duan S. F. Han P. J. Wang Q. M. Liu W. Q. Shi J. Y. Li K.. (2018). The origin and adaptive evolution of domesticated populations of yeast from Far East Asia. Nat. Commun. 9:2690. doi: 10.1038/s41467-018-05106-7, PMID: 30002370 PMC6043522

[ref14] Fan E. Li L. Wu C. Zhang J. You X. Chen P.. (2023). Differences of fungal diversity in Moutai-flavor Daqu under different Koji-making conditions. Food Sci. Technol. 48, 26–33. doi: 10.13684/j.cnki.spkj.2023.05.002

[ref15] Fan E. Yao S. You X. Wu C. Yang J. Yin Y.. (2024). Microbial succession law of the Daqu-making environment in Jiang-flavor Daqu. J. Food Process. Preserv. 2024:2544224. doi: 10.1155/2024/2544224

[ref16] Gou M. Wang H. Yuan H. Zhang W. Tang Y. Kida K. (2015). Characterization of the microbial community in three types of fermentation starters used for Chinese liquor production. J. Inst. Brew. 121, 620–627. doi: 10.1002/jib.272

[ref17] Guan T. Lin Y. Chen K. Ou M. Zhang J. (2020). Physicochemical factors affecting microbiota dynamics during traditional solid-state fermentation of Chinese strong-flavor baijiu. Front. Microbiol. 11:2090. doi: 10.3389/fmicb.2020.02090, PMID: 33013762 PMC7509048

[ref18] Hao F. Tan Y. Lv X. Chen L. Yang F. Wang H.. (2021). Microbial community succession and its environment driving factors during initial fermentation of Maotai-flavor baijiu. Front. Microbiol. 12:669201. doi: 10.3389/fmicb.2021.669201, PMID: 34025626 PMC8139626

[ref19] Hu Y. Huang X. Yang B. Zhang X. Han Y. Chen X.-X.. (2021). Contrasting the microbial community and metabolic profile of three types of light-flavor Daqu. Food Biosci. 44:101395. doi: 10.1016/j.fbio.2021.101395

[ref20] Hu Z. Lin L. Li H. Li P. Weng Y. Zhang C.. (2020). Engineering *Saccharomyces cerevisiae* for production of the valuable monoterpene d-limonene during Chinese baijiu fermentation. J. Ind. Microbiol. Biotechnol. 47, 511–523. doi: 10.1007/s10295-020-02284-6, PMID: 32495196

[ref21] Hu X. Wang K. Chen M. Fan J. Han S. Hou J.. (2020). Profiling the composition and metabolic activities of microbial community in fermented grain for the Chinese strong-flavor baijiu production by using the metatranscriptome, high-throughput 16S rRNA and ITS gene sequencings. Food Res. Int. 138:109765. doi: 10.1016/j.foodres.2020.109765, PMID: 33292946

[ref22] Hu S. Zhu Q. Y. Zhu H. Y. Liu J. Y. Shi Y. Qiu Y. J.. (2025). Yeast diversity in traditional fermented foods of ethnic minorities in China, with the descriptions of four new yeast species. IMA Fungus 16:e146163. doi: 10.3897/imafungus.16.146163, PMID: 40678360 PMC12268275

[ref23] Huang X. Fan Y. Lu T. Kang J. Pang X. Han B.. (2020). Composition and metabolic functions of the microbiome in fermented grain during light-flavor baijiu fermentation. Microorganisms 8:1281. doi: 10.3390/microorganisms8091281, PMID: 32842618 PMC7564364

[ref24] Kang J. Hu Y. Jia L. Zhang M. Zhang Z. Huang X.. (2022). Response of microbial community assembly and succession pattern to abiotic factors during the second round of light-flavor baijiu fermentation. Food Res. Int. 162:111915. doi: 10.1016/j.foodres.2022.111915, PMID: 36461181

[ref25] Kang J. Huang X. Li R. Zhang Y. Chen X. X. Han B. Z. (2024). Deciphering the core microbes and their interactions in spontaneous baijiu fermentation: a comprehensive review. Food Res. Int. 188:114497. doi: 10.1016/j.foodres.2024.114497, PMID: 38823877

[ref26] Lee T. J. Liu Y. C. Liu W. A. Lin Y. F. Lee H. H. Ke H. M.. (2022). Extensive sampling of *Saccharomyces cerevisiae* in Taiwan reveals ecology and evolution of predomesticated lineages. Genome Res. 32, 864–877. doi: 10.1101/gr.276286.121, PMID: 35361625 PMC9104698

[ref27] Lei X. Zheng J. Zhao D. Qiao Z. An M. Zhang X. (2022). Moniliella aeria sp. nov., a novel yeast isolated from the air of a Wuliangye baijiu-making workshop. IJSEM 72:5464. doi: 10.1099/ijsem.0.005464, PMID: 35861490

[ref28] Li W. Chen S. J. Wang J. H. Zhang C. Y. Shi Y. Guo X. W.. (2018). Genetic engineering to alter carbon flux for various higher alcohol productions by *Saccharomyces cerevisiae* for Chinese baijiu fermentation. Appl. Microbiol. Biotechnol. 102, 1783–1795. doi: 10.1007/s00253-017-8715-5, PMID: 29305698

[ref29] Li Y. Cheng Y. Wang H. Hu X. Wang L. Huang Y. (2022). Diverse structure and characteristics of the fungal community during the different rounds of Jiang-flavoured baijiu production in Moutai town. Lwt 161:113313. doi: 10.1016/j.lwt.2022.113313

[ref30] Li W. Cui D. Y. Wang J. H. Liu X. E. Xu J. Zhou Z.. (2017a). Overexpression of different alcohol acetyltransferase genes with BAT2 deletion in *Saccharomyces cerevisiae* affects acetate esters and higher alcohols. Eur. Food Res. Technol. 244, 555–564. doi: 10.1007/s00217-017-2977-0

[ref31] Li W. Fan G. Fu Z. Wang W. Xu Y. Teng C.. (2020). Effects of fortification of Daqu with various yeasts on microbial community structure and flavor metabolism. Food Res. Int. 129:108837. doi: 10.1016/j.foodres.2019.108837, PMID: 32036879

[ref32] Li H. Li S. Mu Z. Lu S. Ai L. Xia Y.. (2024). Research progress on microbial interaction and functional characteristics of non-*Saccharomyces* yeasts in wine brewing process. Food Ferment. Ind. 50, 313–323. doi: 10.13995/j.cnki.11-1802/ts.037916

[ref33] Li P. Lin W. Liu X. Wang X. Gan X. Luo L.. (2017). Effect of bioaugmented inoculation on microbiota dynamics during solid-state fermentation of Daqu starter using autochthonous of *Bacillus*, *Pediococcus*, *Wickerhamomyces* and *Saccharomycopsis*. Food Microbiol. 61, 83–92. doi: 10.1016/j.fm.2016.09.004, PMID: 27697173

[ref34] Li X. R. Ma E. B. Yan L. Z. Meng H. Du X. W. Zhang S. W.. (2011). Bacterial and fungal diversity in the traditional Chinese liquor fermentation process. Int. J. Food Microbiol. 146, 31–37. doi: 10.1016/j.ijfoodmicro.2011.01.030, PMID: 21377228

[ref35] Li W. Wang J. H. Zhang C. Y. Ma H. X. Xiao D. G. (2017b). Regulation of *Saccharomyces cerevisiae* genetic engineering on the production of acetate esters and higher alcohols during Chinese baijiu fermentation. J. Ind. Microbiol. Biotechnol. 44, 949–960. doi: 10.1007/s10295-017-1907-2, PMID: 28176138

[ref36] Li R. Y. Zheng X. W. Zhang X. Yan Z. Wang X. Y. Han B. Z. (2018). Characterization of bacteria and yeasts isolated from traditional fermentation starter (fen-Daqu) through a (1)H NMR-based metabolomics approach. Food Microbiol. 76, 11–20. doi: 10.1016/j.fm.2018.03.015, PMID: 30166130

[ref37] Liu X. Guo K. Zhang H. (2012). Determination of microbial diversity in Daqu, a fermentation starter culture of Maotai liquor, using nested PCR-denaturing gradient gel electrophoresis. World J. Microbiol. Biotechnol. 28, 2375–2381. doi: 10.1007/s11274-012-1045-y 22806111

[ref38] Liu X. Ma D. Yang C. Yin Q. Liu S. Shen C.. (2023). Microbial community succession patterns and drivers of Luxiang-flavor Jiupei during long fermentation. Front. Microbiol. 14:1109719. doi: 10.3389/fmicb.2023.1109719, PMID: 36846777 PMC9950560

[ref39] Liu H. Tan G. Chen Q. Dong W. Chen P. Cai K.. (2021). Detection of viable and total fungal community in zaopei of Chinese strong-flavor baijiu using PMA combined with qPCR and HTS based on ITS2 region. BMC Microbiol. 21:274. doi: 10.1186/s12866-021-02334-8, PMID: 34625023 PMC8499482

[ref40] Liu S. Zhou Y. Ma D. Zhang S. Dong Y. Zhang X.. (2023). Environment microorganism and mature daqu powder shaped microbial community formation in mechanically strong-flavor daqu. Food Biosci. 52:102467. doi: 10.1016/j.fbio.2023.102467

[ref41] Loegler V. Friedrich A. Schacherer J. (2024). Overview of the *Saccharomyces cerevisiae* population structure through the lens of 3,034 genomes. G3 (Bethesda) 14:jkae245. doi: 10.1093/g3journal/jkae245, PMID: 39559979 PMC11631439

[ref42] Lu X. Wu Q. Zhang Y. Xu Y. (2015). Genomic and transcriptomic analyses of the Chinese Maotai-flavored liquor yeast MT1 revealed its unique multi-carbon co-utilization. BMC Genomics 16:1064. doi: 10.1186/s12864-015-2263-0, PMID: 26666414 PMC4678718

[ref43] Luo L. J. Song L. Han Y. Zhen P. Han D. Y. Zhao X.. (2023). Microbial communities and their correlation with flavor compound formation during the mechanized production of light-flavor baijiu. Food Res. Int. 172:113139. doi: 10.1016/j.foodres.2023.113139, PMID: 37689903

[ref44] Luo A. Yang N. Yang J. Hao J. Zhao J. Shi S.. (2022). Effects of microbial interspecies relationships and physicochemical parameters on volatile flavors in sorghum-based fermented grains during the fermentation of Shanxi light-flavored liquor. Food Sci. Nutr. 11, 1452–1462. doi: 10.1002/fsn3.3185, PMID: 36911827 PMC10002873

[ref45] Ma S. Li Z. Luo H. Sun Y. Li J. Huang D. (2024). Effects of *Saccharomyces cerevisiae* on microbial community structure and function of nong-flavour fermented grains. J. Chinese Inst. Food Sci. Technol. 24, 0071–0012. doi: 10.16429/j.1009-7848.2024.02.007

[ref46] Meng X. Wu Q. Wang L. Wang D. Chen L. Xu Y. (2015). Improving flavor metabolism of *Saccharomyces cerevisiae* by mixed culture with *Bacillus licheniformis* for Chinese Maotai-flavor liquor making. J. Ind. Microbiol. Biotechnol. 42, 1601–1608. doi: 10.1007/s10295-015-1647-0, PMID: 26323612

[ref47] Pan Y. Wang Y. Hao W. Duan C. Wang S. Wei J.. (2022). Metatranscriptomics unravel composition, drivers, and functions of the active microorganisms in light-flavor liquor fermentation. Microbiol. Spectr. 10, e02151–e02121. doi: 10.1128/spectrum.02151-21, PMID: 35638860 PMC9241730

[ref48] Pang Z. Hao J. Li W. Du B. Guo C. Li X.. (2023). Investigation into spatial profile of microbial community dynamics and flavor metabolites during the bioaugmented solid-state fermentation of baijiu. Food Biosci. 56:103292. doi: 10.1016/j.fbio.2023.103292

[ref49] Peris D. Ubbelohde E. J. Kuang M. C. Kominek J. Langdon Q. K. Adams M.. (2023). Macroevolutionary diversity of traits and genomes in the model yeast genus Saccharomyces. Nat. Commun. 14:690. doi: 10.1038/s41467-023-36139-2, PMID: 36755033 PMC9908912

[ref50] Pu S. Zhang Y. Lu N. Shi C. Yan S. (2021). Yeasts from Chinese strong flavour Daqu samples: isolation and evaluation of their potential for fortified Daqu production. AMB Express 11:176. doi: 10.1186/s13568-021-01337-y, PMID: 34952959 PMC8709808

[ref51] Qin F. Li M. Zhang W. Zhao J. Yan J. Zhao D.. (2024). Metabolic differences of volatile flavor compounds in baijiu fermented with different Hongqu. Food Ferment. Ind. 50, 252–258. doi: 10.13995/j.cnki.11-1802/ts.037844

[ref52] Qiu F. Du B. Zhang C. Zhu L. Yan Y. Li W.. (2024a). Effects of *Saccharomyces cerevisiae* on microbial community and flavor metabolites in solid-state fermentation of strong-flavor baijiu. Food Biosci. 59:103925. doi: 10.1016/j.fbio.2024.103925

[ref53] Qiu F. Li W. Chen X. Du B. Li X. Sun B. (2024b). Targeted microbial collaboration to enhance key flavor metabolites by inoculating Clostridium tyrobutyricum and *Saccharomyces cerevisiae* in the strong-flavor baijiu simulated fermentation system. Food Res. Int. 190:114647. doi: 10.1016/j.foodres.2024.114647, PMID: 38945586

[ref54] Qiu F. Li W. Zhang Y. Li H. Chen X. Niu J.. (2025). Effect of *Saccharomyces cerevisiae* inoculation on the co-fermentation of Clostridium kluyveri and *Clostridium tyrobutyricum*: a strategy for controlling acidity and enhancing aroma in strong-flavor baijiu. Int. J. Food Microbiol. 435:111172. doi: 10.1016/j.ijfoodmicro.2025.111172, PMID: 40139101

[ref55] Ren J. Y. Liu G. Chen Y. F. Jiang S. Ma Y. R. Zheng P.. (2020). Enhanced production of ethyl lactate in *Saccharomyces cerevisiae* by genetic modification. J. Agric. Food Chem. 68, 13863–13870. doi: 10.1021/acs.jafc.0c03967, PMID: 33166457

[ref56] Ren H. Sun Y. Yang Y. Li Y. Guo X. Zhang B.. (2024). Unraveling the correlations between microbial communities and metabolic profiles of strong-flavor Jinhui Daqu with different storage periods. Food Microbiol. 121:104497. doi: 10.1016/j.fm.2024.104497, PMID: 38637068

[ref57] Sampaio J. P. Pontes A. (2025). Yeast domestication. Curr. Biol. 35, R575–R586. doi: 10.1016/j.cub.2025.04.056, PMID: 40494312

[ref58] Shi W. Chai L. J. Fang G. Y. Mei J. L. Lu Z. M. Zhang X. J.. (2022). Spatial heterogeneity of the microbiome and metabolome profiles of high-temperature Daqu in the same workshop. Food Res. Int. 156:111298. doi: 10.1016/j.foodres.2022.111298, PMID: 35651059

[ref59] Shi J. H. Xiao Y. P. Li X. R. Ma E. B. Du X. W. Quan Z. X. (2009). Analyses of microbial consortia in the starter of fen liquor. Lett. Appl. Microbiol. 48, 478–485. doi: 10.1111/j.1472-765X.2009.02554.x, PMID: 19187491

[ref60] Song Z. Du H. Nie Y. Xu Y. (2020). Identification of core yeasts and their functions in the fermentation process of Maotai-flavor baijiu. Microbiol. China 47, 3504–3514. doi: 10.13344/j.microbiol.china.191046

[ref61] Song Z. Du H. Zhang M. Nie Y. Xu Y. (2019). Schizo*saccharomyces* pombe can reduce acetic acid produced by baijiu spontaneous fermentation microbiota. Microorganisms 7:606. doi: 10.3390/microorganisms7120606, PMID: 31766775 PMC6956116

[ref62] Song Z. Du H. Zhang Y. Xu Y. (2017). Unraveling Core functional microbiota in traditional solid-state fermentation by high-throughput amplicons and Metatranscriptomics sequencing. Front. Microbiol. 8:1294. doi: 10.3389/fmicb.2017.01294, PMID: 28769888 PMC5509801

[ref63] Su C. Zhang K. Z. Cao X. Z. Yang J. G. (2020). Effects of Saccharomycopsis fibuligera and *Saccharomyces cerevisiae* inoculation on small fermentation starters in Sichuan-style Xiaoqu liquor. Food Res. Int. 137:109425. doi: 10.1016/j.foodres.2020.109425, PMID: 33233107

[ref64] Sun J. Liu W. Zang W. Shen G. Ping W. (2012). Composition and distribution of yeasts at different fermentation stages for Maotai-flavor Chinese liquor production. Acta Microbiol Sin. 52, 1290–1296. Available at: https://actamicro.ijournals.cn/actamicrocn/article/abstract/201210-14, PMID: 23289328

[ref65] Sun C. Wang X. Wang R. Zhang Q. Zhang Y. Zhang Z.. (2025). Construction of high ethyl acetate-producing diploid/ tetraploid *Saccharomyces cerevisiae* through CRISPR/Cas9-mediated mating-type switching. Food Biosci. 64:105965. doi: 10.1016/j.fbio.2025.105965

[ref66] Wang L. (2022). Research trends in Jiang-flavor baijiu fermentation: from fermentation microecology to environmental ecology. J. Food Sci. 87, 1362–1374. doi: 10.1111/1750-3841.16092, PMID: 35275413

[ref67] Wang W. Fan G. Li X. Fu Z. Liang X. Sun B. (2020). Application of Wickerhamomyces anomalus in simulated solid-state fermentation for baijiu production: changes of microbial community structure and flavor metabolism. Front. Microbiol. 11:598758. doi: 10.3389/fmicb.2020.598758, PMID: 33329488 PMC7728721

[ref68] Wang J. Liu Z. Wang X. Zhu J. Xu J. Zhang R.. (2024). Optimizing baijiu fermentation with high-yield ethyl caproate-producing *Candida parapsilosis* strain. Lwt 203:116347. doi: 10.1016/j.lwt.2024.116347

[ref69] Wang C. Shi D. Gong G. (2008). Microorganisms in Daqu: a starter culture of Chinese Maotai-flavor liquor. World J. Microbiol. Biotechnol. 24, 2183–2190. doi: 10.1007/s11274-008-9728-0

[ref70] Wang H. Xu Y. (2019). Microbial succession and metabolite changes during the fermentation of Chinese light aroma-style liquor. J. Inst. Brew. 125, 162–170. doi: 10.1002/jib.544

[ref71] Wang Y. Yun J. Zhou M. Wang Z. Li D. Jia X.. (2023). Exploration and application of *Saccharomyces cerevisiae* NJ002 to improve the fermentative capacity of medium-high temperature Daqu. Bioresour. Technol. Rep. 23:101571. doi: 10.1016/j.biteb.2023.101571

[ref72] Wang Q. Zhang H. Liu X. (2016). Microbial community composition associated with Maotai liquor fermentation. J. Food Sci. 81, M1485–M1494. doi: 10.1111/1750-3841.13319, PMID: 27122124

[ref73] Wang N. Zhang P. Zhou X. Zheng J. Ma Y. Liu C.. (2023). Isolation, identification, and characterization of an acid-tolerant Pichia kudriavzevii and exploration of its acetic acid tolerance mechanism. Fermentation 9:540. doi: 10.3390/fermentation9060540

[ref74] Wei J. Nie Y. Du H. Xu Y. (2023). Serine improves lactic acid stress tolerance and ethanol production in Zygo*saccharomyces* bailii in baijiu fermentation. J. Agric. Food Chem. 71, 20295–20303. doi: 10.1021/acs.jafc.3c06246, PMID: 37929595

[ref75] Wei Y. Zhu H. Wen Z. Guo L. Bai M. Wang D.. (2024). *Starmerella fangiana* f.a. sp. nov., a new ascomycetous yeast species from Daqu-making environment and other sources. Int. J. Syst. Evol. Microbiol. 74:006581. doi: 10.1099/ijsem.0.006581, PMID: 39565723 PMC11578291

[ref76] Wu Q. Chen L. Xu Y. (2013). Yeast community associated with the solid state fermentation of traditional Chinese Maotai-flavor liquor. Int. J. Food Microbiol. 166, 323–330. doi: 10.1016/j.ijfoodmicro.2013.07.003, PMID: 23978339

[ref77] Wu Q. Chen B. Xu Y. (2015). Regulating yeast flavor metabolism by controlling saccharification reaction rate in simultaneous saccharification and fermentation of Chinese Maotai-flavor liquor. Int. J. Food Microbiol. 200, 39–46. doi: 10.1016/j.ijfoodmicro.2015.01.012, PMID: 25676241

[ref78] Wu S. Du H. Xu Y. (2023). Daqu microbiota adaptability to altered temperature determines the formation of characteristic compounds. Int. J. Food Microbiol. 385:109995. doi: 10.1016/j.ijfoodmicro.2022.109995, PMID: 36368058

[ref79] Wu Q. Xu Y. Chen L. (2012). Diversity of yeast species during fermentative process contributing to Chinese Maotai-flavour liquor making. Lett. Appl. Microbiol. 55, 301–307. doi: 10.1111/j.1472-765X.2012.03294.x, PMID: 22862564

[ref80] Xiang F. Cai W. Hou Q. Gai J. Dong X. Li L.. (2023). Comparative analysis of the microbial community structure in light-flavor *Daqu* in Taiyuan and Suizhou regions, China. LWT 177:114599. doi: 10.1016/j.lwt.2023.114599

[ref81] Xu Y. Zhi Y. Wu Q. Du R. Xu Y. (2017). Zygo*saccharomyces* bailii is a potential producer of various flavor compounds in Chinese Maotai-flavor liquor fermentation. Front. Microbiol. 8:2609. doi: 10.3389/fmicb.2017.02609, PMID: 29312273 PMC5744019

[ref82] Xue T. Zhang J. Wang T. Bai B. Hou Z. Cheng J.. (2023). Reveal the microbial communities and functional prediction during the fermentation of fen-flavor baijiu via metagenome combining amplicon sequencing. Ann. Microbiol. 73:13. doi: 10.1186/s13213-023-01719-6, PMID: 40761590

[ref83] Yan S. Chen X. Guang J. (2019b). Bacterial and fungal diversity in the traditional Chinese strong flavour liquor Daqu. J. Inst. Brew. 125, 443–452. doi: 10.1002/jib.574

[ref84] Yan S. Tong Q. Guang J. (2019a). Yeast dynamics and changes in volatile compounds during the fermentation of the traditional Chinese strong-flavor Daqu. Lwt 106, 57–63. doi: 10.1016/j.lwt.2019.02.058

[ref85] Yang J. Dou X. Ma Y. (2018). Diversity and dynamic succession of microorganisms during Daqu preparation for Luzhou-flavour liquor using second-generation sequencing technology. J. Inst. Brew. 124, 498–507. doi: 10.1002/jib.528

[ref86] Yang R. Liu P. Chang X. Xu J. Yin H. Fan G.. (2022). Optimization of fermentation conditions for production of ethyl caproate in baijiu using a selected isolate of *Saccharomyces cerevisiae*. Emir. J. Food Agricult. 34, 59–69. doi: 10.9755/ejfa.2022.v34.i1.2808

[ref87] Yao S. Wang P. Bai F. Yu X. Cao Y. Chen K.. (2022). Research on the inventory of microbial food cultures in Chinese traditional fermented foods (2nd edition). Food Ferment. Ind. 48, 272–285. doi: 10.13995/j.cnki.11-1802/ts.029332

[ref88] Yi Z. Jin Y. Xiao Y. Chen L. Tan L. Du A.. (2019). Unraveling the contribution of high temperature stage to Jiang-flavor Daqu, a liquor starter for production of Chinese Jiang-flavor baijiu, with special reference to Metatranscriptomics. Front. Microbiol. 10:472. doi: 10.3389/fmicb.2019.00472, PMID: 30930875 PMC6423406

[ref89] You L. Zhao D. Zhou R. Tan Y. Wang T. Zheng J. (2021). Distribution and function of dominant yeast species in the fermentation of strong-flavor baijiu. World J. Microbiol. Biotechnol. 37:26. doi: 10.1007/s11274-020-02988-y, PMID: 33427975

[ref90] Zha M. Yin S. Sun B. Wang X. Wang C. (2017). STR3 and CYS3 contribute to 2-Furfurylthiol biosynthesis in Chinese sesame-flavored baijiu yeast. J. Agric. Food Chem. 65, 5503–5511. doi: 10.1021/acs.jafc.7b01359, PMID: 28603986

[ref91] Zhang Y. Sun Q. Liu X. Basit R. A. Ma J. Fu Z.. (2024). Screening, identification, and fermentation condition optimization of a high-yield 3-Methylthiopropanol yeast and its aroma-producing characteristics. Foods 13:418. doi: 10.3390/foods13030418, PMID: 38338553 PMC10855053

[ref92] Zhang M. Wu X. Mu D. Xu B. Xu X. Chang Q.. (2021). Profiling the influence of physicochemical parameters on the microbial community and flavor substances of zaopei. J. Sci. Food Agric. 101, 6300–6310. doi: 10.1002/jsfa.11299, PMID: 33969489

[ref93] Zhao H. Du B. Zhao P. Chen X. Zhao J. Wu Q.. (2024). Analysis of ester-producing performance in high-yield ethyl hexanoate yeast and the effect on metabolites in bio- enhanced Daqu, the starter for baijiu and other traditional fermented foods. J. Appl. Microbiol. 135:lxae081. doi: 10.1093/jambio/lxae081, PMID: 38565314

[ref94] Zhen P. Zhu H. Y. Han Y. Luo L. J. Guo L. C. Hu S.. (2025). Ascoidea xinghuacunensis sp. nov., a novel ascomycetous yeast species from Xinghuacun Fenjiu old workshop, Shanxi province of China. IJSEM 75:006700. doi: 10.1099/ijsem.0.006700, PMID: 40029285 PMC11876787

[ref95] Zheng Y. Liang F. Wu Y. Ban S. Huang H. Xu Y.. (2023). Unraveling multifunction of low-temperature Daqu in simultaneous saccharification and fermentation of Chinese light aroma type liquor. Int. J. Food Microbiol. 397:110202. doi: 10.1016/j.ijfoodmicro.2023.110202, PMID: 37086526

[ref96] Zheng X. W. Yan Z. Han B. Z. Zwietering M. H. Samson R. A. Boekhout T.. (2012). Complex microbiota of a Chinese “fen” liquor fermentation starter (fen-Daqu), revealed by culture-dependent and culture-independent methods. Food Microbiol. 31, 293–300. doi: 10.1016/j.fm.2012.03.008, PMID: 22608236

[ref97] Zheng X. W. Yan Z. Nout M. J. Smid E. J. Zwietering M. H. Boekhout T.. (2014). Microbiota dynamics related to environmental conditions during the fermentative production of fen-Daqu, a Chinese industrial fermentation starter. Int. J. Food Microbiol. 182-183, 57–62. doi: 10.1016/j.ijfoodmicro.2014.05.008, PMID: 24863368

[ref98] Zhou J. Li X. Li S. Ding H. Lang Y. Xu P.. (2024). Airborne microorganisms and key environmental factors shaping their community patterns in the core production area of the Maotai-flavor baijiu. Sci. Total Environ. 912:169010. doi: 10.1016/j.scitotenv.2023.169010, PMID: 38040348

[ref100] Zhu C. Cheng Y. Shi Q. Ge X. Yang Y. Huang Y. (2023). Metagenomic analyses reveal microbial communities and functional differences between Daqu from seven provinces. Food Res. Int. 172:113076. doi: 10.1016/j.foodres.2023.113076, PMID: 37689857

